# Progressive osseous heteroplasia in a 10-year-old male child

**DOI:** 10.4103/0019-5413.80050

**Published:** 2011

**Authors:** Girish K Singh, Vikas Verma

**Affiliations:** Department of Orthopaedics, CSM Medical University, Lucknow, Uttar Pradesh, India

**Keywords:** GNAS1 mutation, heterotopic ossification, subcutaneous ossification

## Abstract

We report a sporadic case of progressive osseous heteroplasia (POH) in a 10-year-old male child who developed progressive ossification of the skin and deep connective tissue. The condition needs to be distinguished from other causes of childhood heterotopic ossification, such as fibrodysplasia ossificans progressiva, pseudohypoparathyroidism, and pseudopseudohypoparathyroidism. The cause of POH is an inactivating GNAS1 (guanine nucleotide-binding protein alpha-stimulating activity polypeptide 1) mutation caused only by paternal inheritance of the mutant allele. Most cases are sporadic and only 2 instances of familial transmission have been documented, suggesting an autosomal dominant mode of inheritance with possible somatic mosaicism. The condition is associated with progressive superficial to deep ossification, progressive restriction of range of motion, bleak prognosis, and recurrence if excised.

## INTRODUCTON

Progressive osseous heteroplasia (POH) is a rare autosomal dominant disorder of mesenchymal differentiation characterized by dermal ossification beginning in infancy, followed by increasing and extensive bone formation in deep muscle and fascia.^1^ Most cases are sporadic and only 2 instances of familial transmission^2^,^3^ have been documented, suggesting an autosomal dominant mode of inheritance with possible somatic mosaicism. The molecular defect causing POH is the same as that causing Pseudopseudohypoparathyroidism (PPHP): an inactivating guanine nucleotide binding protein alpha-stimulating activity polypeptide 1(GNAS1 -) mutation caused only by paternal inheritance of the mutant allele. However, patients with PPHP have a constellation of physical findings referred to as Albright hereditary osteodystrophy (AHO), which is not seen in patients with POH. Bastepe and Juppner suggested that POH may be an extreme end of the spectrum of the AHO features seen in PPHP.^4^

## CASE REPORT

**Figure 1:**
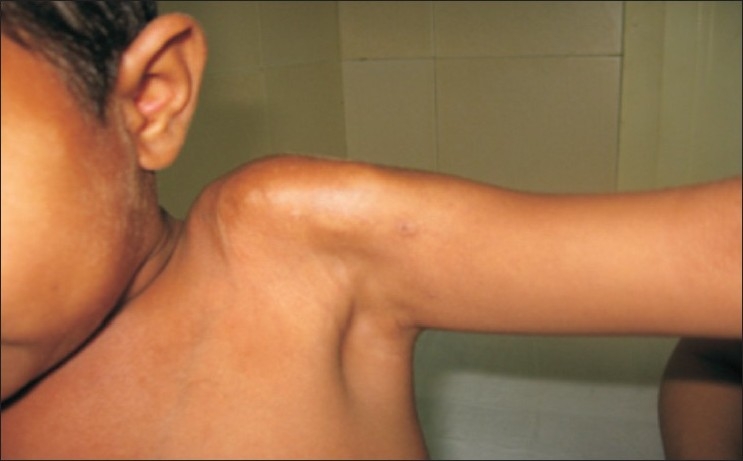
Clinical photograph showing hard cutaneous coalesced plaques over the left shoulder

**Figure 2:**
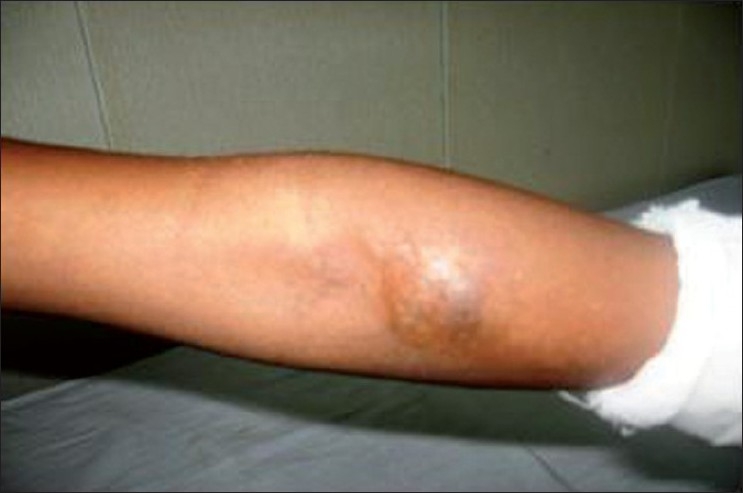
Clinical photograph showing hard cutaneous coalesced plaques over the left elbow

A 10 year old child presented with restricted movements of shoulder, elbow and wrist. The child was delivered at full term without any congenital abnormalities, developed hard cutaneous lesions in the region of his shoulder at the age of 3 months. These lesions coalesced into plaques and spread rapidly over whole of his shoulder and upper arm as shown in Figure 1. Two more similar lesions developed, one over his forearm and the other over his wrist as shown in Figure 2. The lesions have continued to increase in size. No similar condition was found in parents and siblings of the child. The skin over the swellings was fixed and normal in temperature. Movements at shoulder, elbow, and wrist were grossly restricted. The child had normal facies, great toes, thumbs, fifth finger, cervical vertebrae, femoral necks, hearing, scalp hair, and intelligence quotient. He was nonobese and had normal height for his age.

**Figure 3:**
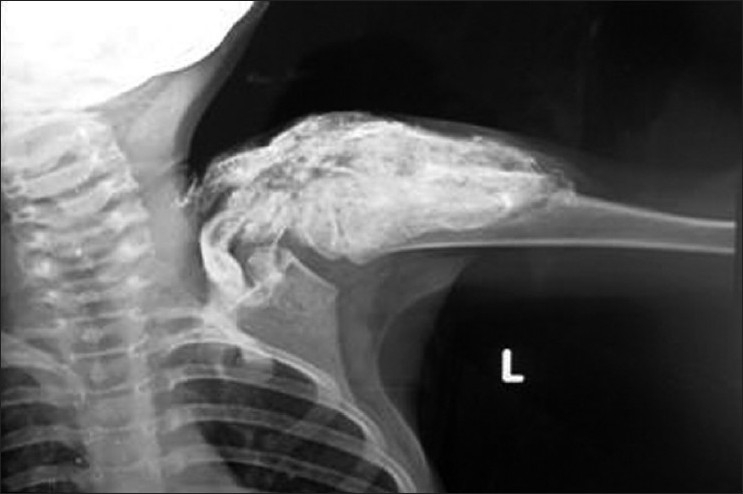
X-ray of left arm with shoulder joint anteroposterior view showing diffuse cocoon-like web of heterotopic bone

**Figure 4:**
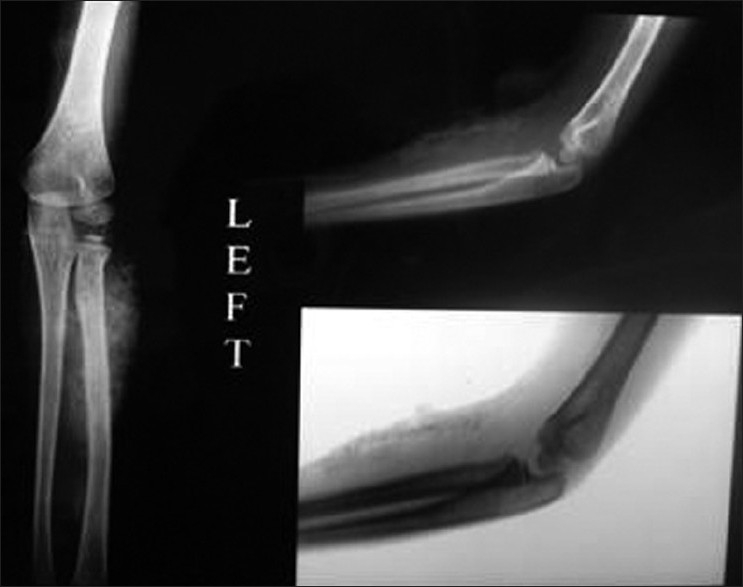
X-ray of left forearm with elbow joint (anteroposterior and lateral views) showing ossification in the dermis and subcutaneous tissue in February 2010

**Figure 5:**
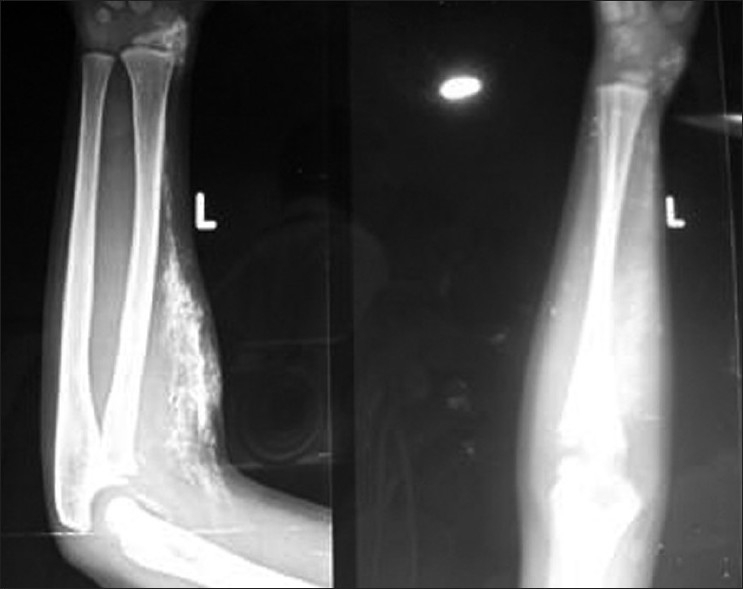
X-rays of forearm with elbow joint (anteroposterior and lateral views) showing ossification in the dermis, subcutaneous tissue, and muscles

Radiologic examination of shoulder revealed a cocoon-like web of heterotopic bone entangling the soft connective tissues from the dermis down through the skeletal muscle without regard to tissue planes as shown in Figure 3. Radiologic examination of elbow and forearm in February 2010 revealed ossification in the dermis and subcutaneous tissue, whereas the muscles seemed to be normal as shown in Figure 4. Repeat radiologic examination of elbow and forearm in April 2010 revealed ossification in dermis, subcutaneous tissue, and muscles as shown in Figure 5. Serum and urine levels of calcium, phosphorus, parathyroid hormone, Vitamin D metabolites, liver function tests, and creatine kinase and aldolase levels were within the normal range. Diagnostic skin and muscle biopsies were performed, which revealed extensive areas of ossification in subcutaneous tissue, dermis, and deltoid in the swelling over the shoulder. Biopsies of skin and muscle of the forearm swelling revealed extensive ossification in the subcutaneous tissue, dermis, and the underlying muscle.

The lesions were not removed taking into account the bleak prognosis associated with surgical removal and skin grafting. The patient was advised physiotherapy to maintain range of motion and the parents were counseled regarding the prognosis of the disease.

## DISCUSSION

The term “progressive osseous heteroplasia” was coined by Kaplan *et al*. to describe a condition characterized by progressive osseous heterotopic ossification (HO) of the skin, subcutaneous fat, and deep connective tissue. POH should be distinguished from other conditions of HO.

Fibrodysplasia ossificans progressiva (FOP) is a rare disorder characterized by physical handicap due to intermittently progressive ectopic ossification and malformed valgoid short big toes, which are often monophalangic. Occasional features include short thumbs, fifth finger clinodactyly, malformed cervical vertebrae, short broad femoral necks, deafness, scalp baldness, and mild mental retardation. The first “flare-up” that leads to the formation of FOP bones is usually before the age of 10 years. The bone growth in FOP starts from the top downward just as bones grow on fetuses, that is, starting on the neck, then shoulders, arms, chest area, and finally on the feet. POH can be distinguished from FOP by the presence of cutaneous ossification, the absence of congenital malformations of the skeleton, the absence of inflammatory tumor-like swellings, the asymmetric mosaic distribution of lesions, the absence of predictable regional patterns of HO, and the predominance of intramembranous rather than endochondral ossification.

POH can be distinguished from pseudohypoparathyroidism by the progression of HO from the skin and subcutaneous tissue into the skeletal muscle, the presence of normal endocrine function, and the absence of a distinctive habitus associated with AHO. AHO is typically associated with dysmorphic “moon” facies, obesity, short stature, brachydactyly, and end-organ resistance to parathyroid hormone. POH can be distinguished from pseudopseudohypoparathyroidism, a condition of subcutaneous ossification without hormone resistance, by the absence of physical findings characteristic of AHO.^5^

Shore *et al*.^6^ tested the hypothesis that GNAS1 mutations cause POH in 18 patients with sporadic or familial POH. They identified heterozygous inactivating GNAS1 mutations in 13 of the 18 probands with POH. The defective allele in POH was inherited exclusively from fathers, a result consistent with a model of imprinting for GNAS1. Direct evidence that the same mutation can cause either POH or AHO was observed within a single family, in which the phenotype correlated with the parental origin of the mutant allele. They concluded that paternally inherited inactivating GNAS1 mutations cause POH. Faust *et al*^7^ identified a heterozygous germline mutation in the GNAS1 gene in an Albanian girl with POH of the face.

Adegbite *et al*^8^ reviewed the charts of 111 individuals who had cutaneous and subcutaneous ossification for 8 characteristics: age of onset of HO, presence and location of HO, depth of HO, type of HO, progression of HO, features of AHO, parathyroid hormone resistance, and GNAS mutation analysis. They found, based on clinical criteria that POH and progressive HO syndromes are at the severe end of a phenotypic spectrum of GNAS-inactivating conditions associated with extraskeletal ossification. They found that most individuals with superficial or progressive ossification had mutations in GNAS, but there were no specific genotype-phenotype correlations that distinguished the more progressive forms of HO (eg, POH) from the nonprogressive forms (osteoma cutis, AHO, and PHP1a/c).

The treatment of POH is very disappointing. Surgical removal has lead to recurrence in most patients. The only treatment available, although limited, is physiotherapy to preserve movement and prevention of skin breakdown. The parents need to be counseled to continue education as the child is mentally normal and disease does not seem to progress rapidly in adults.
